# Supplementary material for: Causal Relationship between Aging and Anorexia Nervosa: A White-Matter-Microstructure-Mediated Mendelian Randomization Analysis
Source: Biomedicines. 2024 Aug 16;12(8):1874. doi: 10.3390/biomedicines12081874 (PMC11351342; doi:10.3390/biomedicines12081874)
Supplement: Supplementary file 1 [file biomedicines-12-01874-s001.zip › Supplementary Figure/Figure S8/Leaveoneout plot of MR analysis for TL to ubm-b-1991.pdf]

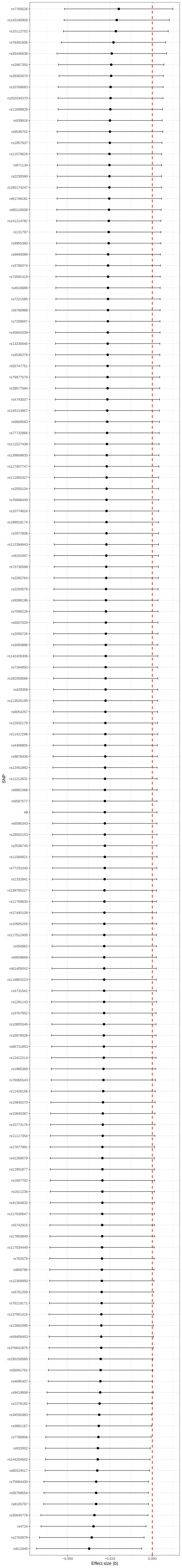
rs7705526
rs143190905
rs10112752
rs78491606
rs35446936
rs2967355
rs28363070
rs10768683
rs202034370
rs11699829
rs939916
rs6536702
rs1957937
rs11579626
rs871134
rs2230590
rs185174247
rs61748181
rs80116508
rs141214782
rs131797
rs9955360
rs9940099
rs3785074
rs73581419
rs4616688
rs7221585
rs6790988
rs7209057
rs45604339
rs13230646
rs4530278
rs55747751
rs79977579
rs28577594
rs4743037
rs145114957
rs6669563
rs77732966
rs111527438
rs139669835
rs117407747
rs111950327
rs2555104
rs76666449
rs10774624
rs188918174
rs2977608
rs112394943
rs8102497
rs73730598
rs2282764
rs2293579
rs9398196
rs7099229
rs6007020
rs2056726
rs3093888
rs142426306
rs7164950
rs182059586
rs429358
rs113525195
rs6054257
rs12932179
rs11412296
rs4498805
rs9878436
rs12451892
rs11212631
rs6881568
rs6587577
All
rs6590343
rs28502153
rs2538745
rs11584821
rs77231040
rs1332941
rs139795227
rs11769630
rs17445108
rs10905255
rs117512405
rs450962
rs6659669
rs61405042
rs116863222
rs4731541
rs1291143
rs3767952
rs10805346
rs16978028
rs66731853
rs12412214
rs1985369
rs76065543
rs11428158
rs10840270
rs10845387
rs10773176
rs11117354
rs17677991
rs41269079
rs11991877
rs1907702
rs1611236
rs41304832
rs117630647
rs5742915
rs17803849
rs117034449
rs762679
rs869785
rs12369950
rs6751209
rs76219171
rs137901416
rs13062095
rs59409453
rs76641875
rs150150565
rs56061761
rs4695407
rs9419958
rs2276182
rs34550383
rs3891167
rs7790856
rs932002
rs144204502
rs80324517
rs75664430
rs56799554
rs8105767
rs35640778
rs4724
rs2763979
rs611646
SNP
-0.050
-0.025
0.000
Effect size (b)
